# Books and Magazines

**Published:** 1905-03

**Authors:** 


### Books and Magazines.

DIET IN HEALTH AND DISEASE. By Julius Friedenwald, M. D., Clinical Professor of Diseases of the Stomach in the College of Physicians and Surgeons, Baltimore; and John Ruhräh, M. D., Clinical Professor of Diseases of Children in the College of Physicians and Surgeons, Baltimore. Octavo volume of 689 pages. Philadelphia, New York, London: W. B. Saunders & Company, 1904. Cloth, $4.00 net.

This latest work on diet is practical and comprehensive, prepared to meet the needs of the general practitioner, medical student, hospital interne, and trained nurse. It contains a full account of food stuffs, their uses and chemical compositions. Dietic management in all diseases in which diet plays a part in treatment is carefully considered, the articles on diet in diseases of the digestive organs containing numerous diet lists and explicit instructions for administering. The feeding of infants and children, of patients before and after anesthesia and surgical operations, and the latest methods for feeding after gastro-intestinal operations have never before been discussed with such practical detail. The subject of rectal enemata is given completely, with recipes and full instructions as to technic. Diet is considered in its relations to age, occupation, and environment and the beneficial results from the rest cure have been accorded prominent consideration. There is also a section on food adulteration and the resultant diseases. Withal, this is a work well worthy the reputation of its authors, and we most cheerfully recommend it.

A TEXT-BOOK OF LEGAL MEDICINE. By Frank Winthrop Draper, A. M., M. D., Professor of Legal Medicine in Harvard University; Medical Examiner for the County of Suffolk, Massachusetts. Octavo volume of 573 pages, fully illustrated. Philadelphia, New York, London: W. B. Saunders & Company, 1905. Cloth, $4.00 net.

The subject of Legal Medicine is one of great importance, especially to the general practitioner, for it is to him that calls to attend cases which may prove to be medicolegal in character most frequently come. Dr. Draper has written his work both for the general practitioner and for the medical student. He has not only cited illustrative cases from standard treatises on forensic medicine, but these he has supplemented with details from his own exceptionally full experience—an experience gained during his service as Medical Examiner for the City of Boston for the past twenty-six years. During this time his investigations have comprised nearly eight thousand deaths under a suspicion of violence. The author's long teaching career has enabled him to state facts and detail procedures with a clearness rarely met in a work on Legal Medicine. Withal, we think Dr. Draper's book is unusually satisfactory, it is more—it surpasses our expectations.

---

The Practical Application of the Roentgen Rays in Therapeutics and Diagnosis. By William Allen Pusey, A. M., M, D., Professor of Dermatology in the University of Illinois; and Eugene W. Caldwell, B. S., Director of the Edward N. Gibbs Memorial X-Ray Laboratory of the University and Bellvue Hospital Medical College, New York. Second Edition thoroughly revised. Handsome octavo volume of 690 pages, with 195 illustrations, including four colored plates. Philadelphia, New York, London: W. B. Saunders & Company, 1904. Cloth, $5.00 net; Sheep or Half Morocco, $6.00 net.

This excellent work has attained the distinction of two large editions in one year—a proof not only that such a work was needed, but also of the book's practical value. The vast amount of literature accumulated during the past year has been very carefully digested, and the latest knowledge and advancements incorporated. A practical feature of the work lies in the fact that nearly all the illustrations represent actual clinical subjects, showing the benefits of the X-rays at various stages of their application. The chapters by Caldwell give full details regarding the use and management of the apparatus, the text being fully illustrated with many photographs and drawings, including four full-page colored plates. The second edition has been brought strictly down to date, especially the case histories cited; and by the addition of much new matter and a number of new illustrations, the usefulness of the work has been greatly extended. It is the latest and best book on this subject.

---

Gallstones and Their Surgical Treatment. By B. G. A. Moynihan, M. S. (Lond.), F. R. C. S., Senior Assistant Sur-

### THE ONE REMEDY

**which experience proves is free from detrimental effects is**

### GRAY'S Glycerine TONIC Comp.

**Try it in convalescence, respiratory disorders, anaemia, malnutrition, nervous exhaustion.**

**THE PURDUE FREDERICK CO.,**
**298 Broadway, New York**

geon to Leeds General Infirmary, England. Octavo volume of 386 pages, illustrated with text-cuts, some in colors, and nine colored insert plates. Philadelphia, New York, London: W. B. Saunders & Co., 1904. Cloth, $4, net.

The great and increasing importance of the subject of gallstone disease is a sufficient warrant for the publication of this forelying work, and Mr. Moynihan's extensive experience in treating cholelithiasis specially fits him to write an authoritative and trustworthy work such as we have found this. A full account is given of the origin and causation of gallstones, and of the pathologic changes and clinical manifestations to which they give rise. Special attention has been paid to the detailed description of the early symptoms of cholelithiasis, enabling a diagnosis to be made in the stage in which surgical treatment can be most safely adopted. Every phase of the gallstone disease is dealt with, and is illustrated by a large number of clinical records. The account of the operative treatment of all forms and complications is full and accurate. The beautiful illustrations, a number of which are in color, including nine insert plates, are unusually clear and artistic, and form a special feature. We know of no book on the same subject that can in any way compare with Mr. Moynihan's work.

---

BACTERIOLOGY AND SURGICAL TECHNIC FOR NURSES. By Emily M. A. Stoney, Superintendent of the Training School for Nurses, St. Anthony's Hospital, Rock Island, Ill. Second edition, thoroughly revised and much enlarged by Frederick R. Griffith, M. D., Surgeon, Fellow of the New York Academy of Medicine. 12mo volume of 278 pages, fully illustrated. Philadelphia, New York, London: W. B. Saunders & Co., 1905. Cloth, $1.50, net.

The revision for the second edition of this practical work has been most thorough and extensive, the book having been increased in size by the addition of over eighty pages and many cuts. Dr. Frederic R. Griffith, to whom the work of revision was intrusted, has wisely added several chapters of unquestionable importance, namely: Bandaging and Dressing; Obstetric Nursing, Care of Infants, etc.; Hygiene and Personal Conduct of the Nurse, etc. Nurses will find the glossary at the back of much value. As a whole we think it a compact, useful book, pregnant with just the information that nurses most and constantly need.

---

SAUNDERS' MEDICAL HAND-ATLAS AND EPITOME OF OPERATIVE OPHTHALMOLOGY. By Dr. O. Haab, of Zürich. Edited, with additions, by George E. de Schweinitz, M. D., Professor of Ophthalmology in the University of Pennsylvania. With thirty colored lithographic plates, 154 text-cuts, and 377 pages of text. Philadelphia, New York, London: W. B. Saunders & Co., 1905. Cloth, $3.50, net.

This new volume forms an admirable conclusion of the series of atlases on the eye prepared by Professor Haab. Beginning with a thorough discussion of the proper construction of operating-rooms, narcosis, sterilization as applied to ophthalmic instruments, and disinfection, ophthalmic operations are described with all the fidelity and clearness that thirty years' conscientious practice in eye work naturally brings. The colored illustrations exhibit the same perfection of art and accurateness of detail which we have found only in this invaluable series of atlases. We note that the able editor, Dr de Schweinitz, has rendered the volume much more valuable by his many additions throughout the text. Any one interested in eye work will find this book of more value than any other volume recently published.

Diseases of the Liver, Gall-Bladder, and Bile-Ducts. By H. D. Rolleston, A. M., M. D. (Cantab.), F. R. C. P., Physician to St. George's Hospital, London; formerly Examiner in Medicine at the University of Durham, England. Octavo volume of 794 pages, fully illustrated, including seven colored insert plates. Philadelphia, New York, London: W. B. Saunders & Co., 1904. Cloth, $6, net.

Dr. Rolleston's new work is undoubtedly the most voluminous treatise on diseases of the liver yet published in English. And more than that, it is destined to become an authority on the subjects of which it treats. The author has for many years made a special study of diseases of the digestive system, and his reputation in the treatment of heptic diseases is sufficient assurance of the practical usefulness of this new work. The text includes all the affections of the liver, completely and clearly discussed, special attention being given to pathology and treatment. A large number of clinical cases are quoted, which will be found of great value to the practitioner in diagnosing individual cases. Besides Diseases on the Liver, the book contains articles on Diseases of the Gall-Bladder and Bile-Ducts, which are equally as trustworthy and authoritative as the section on the liver. The illustrations, both those showing gross appearances and the microphotographs, are unusually excellent, and include seven colored insert plates of great merit. The mechanical appearance of the work is in keeping with the high standard of the text. We certainly most highly recommend it to our readers.

---

Pathological Technique.—A Practical Manual for Workers in Pathological Histology and Bacteriology, including Directions for the Performance of Autopsies and for Clinical Diagnosis by Laboratory Methods. By F. B. Mallory, M. D., Associate Professor of Pathology, and J. H. Wright, M. D., Instructor in Pathology, Harvard University Medical School. Third edition, revised and enlarged. Octavo, 469 pages, with 140 illustrations. Philadelphia, New York, London: W. B. Saunders & Co., 1904. Cloth, $3, net.

The third edition of this successful work keeps pace with the great advances made in pathology, and its value as a laboratory and post-mortem guide remains undisputed.

In subjecting this book to thorough revision the authors have kept in view the needs of the laboratory worker, whether student, practitioner or pathologist, for a practical manual of modern methods in the study of pathological material. Many parts have been rewritten, many new methods have been added, and the number of illustrations has been increased.

---

Atlas and Epitome of General Pathologic Histology. By Dr. H. Dürck, of Munich. Edited, with additions, by Ludvig Hektoen, M. D., Professor of Pathology, Rush Medical College, in affiliation with the University of Chicago. With 172 colored figures on 77 lithographic plates, 36 text-cuts, many in colors, and 371 pages of text. Philadelphia, New York, London: W. B. Saunders & Co., 1904. Cloth, $5, net.

This new atlas in Saunders' Medical Hand-Atlases is indeed a worthy addition to the series. All the accepted views regarding the significance of pathologic processes have been concisely stated, conflicting theories having been unwisely omitted. The illustrations have been made from original specimens without combining different microscopic fields, extraordinary care having been taken to reproduce them as near perfection as possible. In many cases as high as twenty-six colors have been required to reproduce the original painting. In editing the volume, Dr. Hektoen has incorporated much useful matter; and unquestionably this atlas will be as favorably received as the previous volumes on Special Pathologic Histology. In our opinion, it will be found of unusual value to the medical profession generally.

---

A TEXT-BOOK OF PATHOLOGY. By Joseph McFarland, M. D., Professor of Pathology and Bacteriology in the Medico-Chirurgical College of Philadelphia; Pathologist to the Medico-Chirurgical Hospital, Philadelphia. Handsome octavo volume of 818 pages, with 350 illustrations, a number in colors. Philadelphia, New York, London: W. B. Saunders & Co., 1904. Cloth, $5, net; sheep or half Morocco, $6, net.

It was with anticipations of much pleasure and interest that the reviewer began reading Dr. McFarland's work on Pathology, and he can truthfully say that his greatest expectations were more than fulfilled. The book is excellent—excellent as regards both text and illustrations. Of the latter there are a number of beautiful ones in colors, printed directly in the text. Dr. McFarland's thirteen years' experience as a teacher of this subject, besides his extensive personal research in the laboratory, has fitted him most admirably to write a text-book on pathology, and this superb forelying work is all that any one—student or practitioner—could desire. Unlike most works on pathology, the subject is treated, not from the professor's point of view, but from that of the student, the many difficult theories of the science being explained in clear, concise language. A number of works on pathology have come to the reviewer's desk within the last few years, but none has reached the standard of excellence held by Dr. McFarland's work.

---

A MANUAL OF PERSONAL HYGIENE.—Proper Living Upon a Physiologic Basis. By American authors. Edited by Walter L. Pyle, A. M., M. D., Assistant Surgeon to the Wills Eye Hospital, Philadelphia. Second edition, revised and enlarged. 12mo volume of 441 pages, fully illustrated. Philadelphia, New York, London: W. B. Saunders & Co., 1904. Bound in silk, $1.50, net.

A short time ago, when Dr. Pyle's work first appeared, we gave it our unqualified recommendation. The new second edition, just issued, is evidence that this work has filled the need. Personal hygiene is applied physiology, and a proper understanding of certain elemental truths on practical human physiology must first be acquired before it can be applied. Knowledge of the normal functions of the body and simple methods of keeping them in healthy action is the one thing that no educated person should be excused from possessing. The ordinary instructions in physical education, physiology, dietetics, and exercise is not sufficient, and often faulty. Dr. Pyle has selected eight prominent American physicians, each writing upon his chosen specialty, and setting forth the means of health in this "Manual of Personal Hygiene" with a *simplicity, conciseness, and authority* that has never been approached in any similar work.

In this new second edition there have been added, and fully illustrated, chapters on Domestic Hygiene and on Home Gymnastics, besides an Appendix containing methods of Hydrotherapy, Thermotherapy, Mechanotherapy, and First Aid measures in medical and surgical accidents and emergencies. Physicians could render no better service to their patients than the recommendation of this book.

